# Linkage disequilibrium levels and allele frequency distribution in Blanco Orejinegro and Romosinuano Creole cattle using medium density SNP chip data

**DOI:** 10.1590/1678-4685-GMB-2016-0310

**Published:** 2018

**Authors:** Diego Bejarano, Rodrigo Martínez, Carlos Manrique, Luis Miguel Parra, Juan Felipe Rocha, Yolanda Gómez, Yesid Abuabara, Jaime Gallego

**Affiliations:** 1 Corporación Colombiana de Investigación Agropecuaria - Corpoica Corporación Colombiana de Investigación Agropecuaria - Corpoica Centro de Investigación Tibaitatá Cundinamarca Colombia Corporación Colombiana de Investigación Agropecuaria - Corpoica. Centro de Investigación Tibaitatá, Cundinamarca, Colombia; 2 Universidad Nacional de Colombia Universidad Nacional de Colombia Bogotá Colombia Universidad Nacional de Colombia. Bogotá, Colombia; 3 Corporación Colombiana de Investigación Agropecuaria - Corpoica Corporación Colombiana de Investigación Agropecuaria - Corpoica Centro de Investigación Obonuco Nariño Colombia Corporación Colombiana de Investigación Agropecuaria - Corpoica. Centro de Investigación Obonuco, Nariño, Colombia; 4 Corporación Colombiana de Investigación Agropecuaria - Corpoica Corporación Colombiana de Investigación Agropecuaria - Corpoica Centro de Investigación Turipaná Córdoba Colombia Corporación Colombiana de Investigación Agropecuaria - Corpoica. Centro de Investigación Turipaná, Córdoba, Colombia; 5 Corporación Colombiana de Investigación Agropecuaria - Corpoica Corporación Colombiana de Investigación Agropecuaria - Corpoica Centro de Investigación El Nus Antioquia Colombia Corporación Colombiana de Investigación Agropecuaria - Corpoica. Centro de Investigación El Nus, Antioquia, Colombia

**Keywords:** Creole breeds, BovineSNP50, linkage disequilibrium, minor allele frequency

## Abstract

The linkage disequilibrium (LD) between molecular markers affects the accuracy of genome-wide association studies and genomic selection application. High-density genotyping platforms allow identifying the genotype of thousands of single nucleotide polymorphisms (SNPs) distributed throughout the animal genomes, which increases the resolution of LD evaluations. This study evaluated the distribution of minor allele frequencies (MAF) and the level of LD in the Colombian Creole cattle breeds Blanco Orejinegro (BON) and Romosinuano (ROMO) using a medium density SNP panel (BovineSNP50K_v2). The LD decay in these breeds was lower than those reported for other taurine breeds, achieving optimal LD values (r^2^ ≥ 0.3) up to a distance of 70 kb in BON and 100 kb in ROMO, which is possibly associated with the conservation status of these cattle populations and their effective population size. The average MAF for both breeds was 0.27 ± 0.14 with a higher SNP proportion having high MAF values (≥ 0.3). The LD levels and distribution of allele frequencies found in this study suggest that it is possible to have adequate coverage throughout the genome of these breeds using the BovineSNP50K_v2, capturing the effect of most QTL related with productive traits, and ensuring an adequate prediction capacity in genomic analysis.

## Introduction

Gene alleles which are physically close in a chromosome are inherited in an interconnected manner by inheritance properties, and these are not passed to the offspring independently but as blocks of alleles or haplotypes provided by each parent (Ardlie *et al.*, 2002; Pérez O’Brien *et al.*, 2014a). This condition creates a level of correlation among the alleles that is known as linkage disequilibrium (LD), and this concept can be extended to any nucleotide in the genome, as well as to any type of genetic molecular marker, such as single nucleotide polymorphisms (SNP) (Pérez O’Brien *et al.*, 2014a). Therefore, the concept of LD between molecular markers reflects the correlation that exists between the genotypes of two markers or the degree of nonrandom association between their alleles (Porto-Neto *et al.*, 2014). This correlation is mainly derived from the physical proximity, although it may also be influenced by several evolutionary processes and historical events that have occurred in the population (Reich *et al.*, 2001; Ardlie *et al.*, 2002; Khatkar *et al.*, 2008).

Knowledge of the LD decay magnitude and pattern along the bovine genome has important implications on a large number of methodologies based on genomic data that are currently used in genetics and animal breeding, as the genome-wide association studies (GWAS) (Ardlie *et al.*, 2002), prediction and genomic selection (Meuwissen *et al.*, 2001; The Bovine Genome Sequencing and Analysis Consortium *et al.*, 2009), genomic markers imputation (Piccoli *et al.*, 2014), marker-assisted selection (MAS), quantitative trait loci (QTL) mapping, parentage testing, genomic markers-based disease testing, among others. Most of these tools are routinely applied in animal breeding programs, and their implementation success is inherently dependent on the LD levels present between markers, and between markers and their surrounding genomic regions (Pérez O’Brien *et al.*, 2014a).

The LD is the foundation that supports gene mapping and genome-wide association studies (GWAS), which are important tools for the exploration of the genetic basis that regulates the expression of economically important traits in cattle (McKay *et al.*, 2007; Espigolan *et al.*, 2013). Moreover, the LD is also a useful tool for exploring the degree of diversity among races, inferring the crossing-over distribution, and identifying genome regions that have been subject to different selection pressures (McKay *et al.*, 2007; Bohmanova *et al.*, 2010; Porto-Neto *et al.*, 2014). Likewise, high resolution LD maps have also provided useful information for high density SNP design panels that are used in genomic selection (GS) (Matukumalli *et al.*, 2009; Wiggans *et al.*, 2009; Bohmanova *et al.*, 2010).

The level of LD present in different populations and cattle breeds directly affects the results found in GWAS as well as the accuracy of genomic breeding value estimates, since this analysis explores the existing LD among the markers, under the assumption that the effects of chromosomal segments will be the same throughout the population. This assumes that the markers are in LD with the genes that are responsible for the expression of the trait of interest (QTL) (Meuwissen *et al.*, 2001). Therefore, the markers density to be used must be high enough to ensure that all QTLs of interest are in LD with at least one or more markers (Meuwissen *et al.*, 2001; Meuwissen, 2009; Espigolan *et al.*, 2013). Previous studies that used SNP markers to describe the LD decay patterns in cattle at the whole genome level (McKay *et al.*, 2007; Khatkar *et al.*, 2008; Gibbs *et al.*, 2009; Bohmanova *et al.*, 2010; Beghain *et al.*, 2013; Hozé *et al.*, 2013), have suggested that between 30,000 and 300,000 SNPs are required to carry out a GWAS, and this depends on the trait evaluated and the statistical power desired (McKay *et al.*, 2007; Khatkar *et al.*, 2008).

Quantification of the LD extent in the genomes of cattle breeds is a necessary first step to establish if the number of markers included in the genotyping panel is sufficient to obtain good results in QTL mapping by GWAS (Goldstein, 2001; Carlson *et al.*, 2004; McKay *et al.*, 2007), and to implement genomic selection strategies (Meuwissen *et al.*, 2001; Khatkar *et al.*, 2008; Sargolzaei *et al.*, 2008; Qanbari *et al.*, 2010). However, its importance is often neglected, creating biases in any analysis performed. For this reason, the aim of this study was to evaluate the distribution of minor allele frequencies (MAF) and the LD level across the genome in two Colombian Creole cattle breeds, using a medium density SNP panel (50K).

## Materials and Methods

### Animals and genotypes

A total of 866 individuals of the Creole cattle breeds Blanco Orejinegro (BON) (n=500) and Romosinuano (ROMO) (n=366) were used in this study. These were genotyped for 54,609 SNPs using the BeadChip BovineSNP50K_v2 (Illumina Inc., 2016), and DNA samples were extracted from blood or semen and genotyped at the Molecular Genetics Laboratory of Corpoica, Tibaitatá Research Center, following the Infinium HD Assay Protocol (Illumina Inc., San Diego, CA, USA). To obtain the genotypes each chip was scanned using HiScan®, and then the data base was analyzed using the Illumina GenomeStudio software (Illumina Inc.).

### Genotype quality control

Quality control (QC) procedures were applied to SNP genotype data independently for each racial group according to the rules described by Wiggans *et al.* (2010), using the PLINK v1.9 software (http://pngu.mgh.harvard.edu/purcell/plink/) (Purcell *et al.*, 2007). The SNP data was removed either when their call rate was less than 90%, when these had pedigree errors, or when they showed an extreme departure from the Hardy-Weinberg equilibrium (e.g., SNPs on autosomal chromosomes with both homozygous genotypes and no observation of heterozygotes). Additionally, the SNPs were also removed when these had unknown genomic positions, if they were located on sex chromosomes, and if the MAF was lower than or equal to 0.03.

### Minor allele frequency estimation

After performing QC, PLINK was used to estimate the minor allele frequency (MAF) for all autosomal markers included in the two data sets. According to the distribution of allele frequencies, five categories were established representing the proportion of SNPs with MAF values that fall within the following ranges: ≥ 0.03 to < 0.1, ≥ 0.1 to < 0.2, ≥ 0.2 to < 0.3, ≥ 0.3 to < 0.4 and ≥ 0.4 to ≤ 0.5. Note that values that fell less than 0.03 were eliminated. The results of the comparison between the two breeds were plotted using R.

### Linkage disequilibrium (LD) analysis

The square of the correlation coefficient between two loci (r^2^) was used as the LD measure, indicating the ability of the alleles present in a marker to predict the presence of alleles on a second marker located on a certain genetic distance measured in base pairs (Ke *et al.*, 2004). The r^2^ value based on the genotype frequencies of each SNP was coded as 0, 1, or 2 copies of allele B, and was calculated using PLINK (Purcell *et al.*, 2007). This was carried out for all possible combinations of SNP pairs separated by a maximum distance of up to 1 mega base (Mb) within each chromosome. Using the values of r^2^ ≥ 0.1, the LD decay was analyzed for two different maximum distances between SNP pairs, defined as ≤200 kb and ≤1 Mb. To show the average trend in the LD decay, r2 was calculated for all the possible SNP comparisons included within the different size windows, i.e. 1 kb, 10 kb and 100 kb, depending on the distance between markers. The trend in the LD decay for each breed was plotted through the whole genome and by chromosome (BTA).

## Results

**Figure 1:**
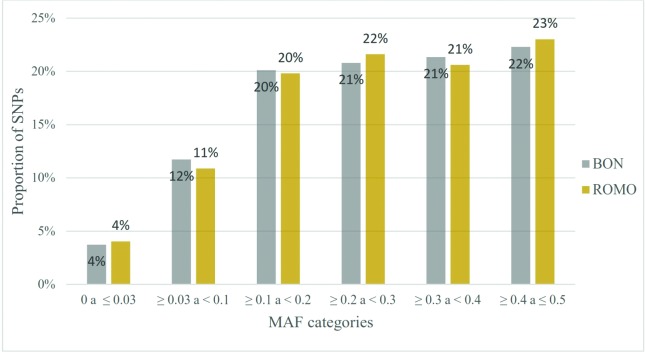
Minor Allele Frequencies (MAF) distribution for each breed. Proportion of SNPs (Y axis) included in the Bovine SNP50K v2 (Illumina Inc. 2016), found within each category of MAF (X axis) for each breed.

A total of 40,555 autosomal SNPs were identified after carrying out quality control on BON cattle (74.26%), and 40,421 autosomal SNP on ROMO cattle (74.02%). The distribution of MAF shows a similar trend in both breeds. A higher proportion of SNPs had high MAF values with approximately 45% of the SNPs falling into the last two categories (MAF ≥ 0.3), and a lower percentage were classified in the first categories, with 32 to 33% of the SNPs showing a MAF value lower than 0.2 (Figure 1).

**Figure 2:**
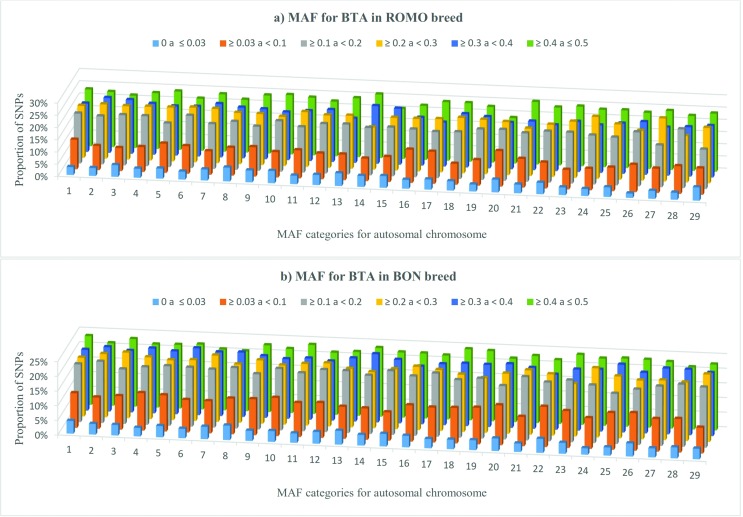
Proportion of SNPs for various categories of minor allele frequencies (MAF) calculated for each autosomal chromosome in ROMO (a) and BON (b) breeds.

Overall, all chromosomes followed the same trend in both breeds with a higher proportion of SNPs located in the last two categories (MAF ≥ 0.3) (Figure 2a,b). In the case of BON, chromosomes BTA18, BTA19 and BTA23 showed a higher SNPs proportion in the last category (MAF ≥ 0.4). In ROMO, chromosomes with a larger number of SNPs with MAF ≥ 0.4, were BTA14 (27.2%), BTA21 (26.5%) and BTA10 (25.6%). With regards to the category that includes SNPs with ≥ 0.03 and < 0.1 values, chromosomes with a higher SNPs proportion in BON were BTA20 (15.4%), BTA16 (14.2%) and BTA17 (13.7%). In ROMO, chromosomes with the largest number of SNPs with MAF values of less than 0.1 were BTA22 (14.4%) and BTA20 (14.2%).

After attaining autosomal SNPs through a QC process, the level of LD between autosomal markers was assessed using a correlation coefficient between two loci (r^2^). In order to consider all possible SNP pairs with a distance of less than or equal to 100 kb between markers, 28,830 SNP combinations pairs were obtained to estimate LD across the 29 autosomes in the BON breed, and 28,924 SNP combinations pairs in the ROMO breed. Up to a distance of ≤1 Mb, the number of combinations of SNP pairs (r^2^ ≥ 0.1) increased to 177,395 in the BON breed and to 222,480 in the ROMO breed.

**Figure 3:**
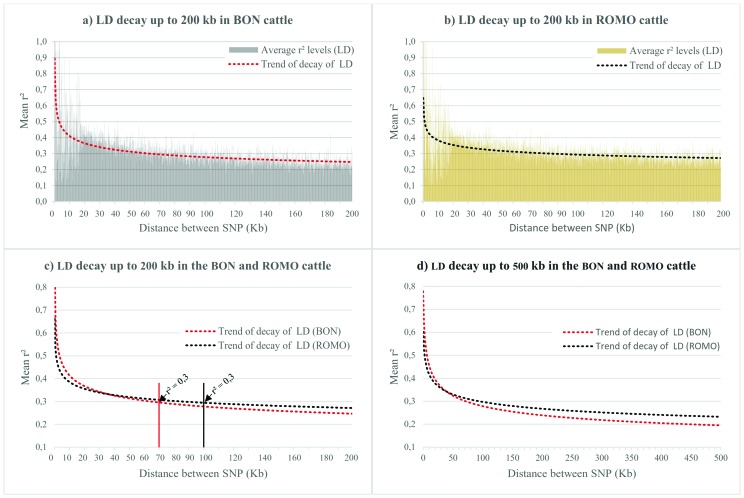
LD decay (r^2^) from 0 up to 200 Kb in BON (a) and ROMO (b) breeds, comparison between breeds (c) and LD decay (r^2^) from 0 up to 500 Kb in both breeds (d).

The LD decay analysis of up to 200 kb using 1 kb windows is shown in Figure 3. For both breeds the LD starts at a high level with r^2^ levels > 0.9 in the first windows (1 kb), followed by a rapid decline, with a quite variable trend of up to 20 kb, reaching at this point an average r^2^ levels of 0.38 in the ROMO breed and an average of 0.41 in the BON breed. From this distance the LD decay pattern becomes more stable and follows a more defined trend. At a distance of 200 kb the mean r^2^ found was 0.27 for the ROMO breed and 0.25 for the BON breed (Figure 3c). Moreover, when comparing the LD decay between breeds, at short distances of less than 40 kb, the decay is faster in the ROMO breed than in the BON breed, but from this point onwards, a greater LD breakdown is seen in the BON breed compared with the ROMO breed that has a higher level of LD from 40 kb (Figure 3c).

In the LD decay analysis for distances greater than 100 kb and up to 1 Mb and using 100 kb windows, a decreasing trend in the mean r^2^ was found (Figure 3d), but without showing a sharp decay with the increasing physical distance between markers. For the ROMO breed, between 100 Kb and 200 Kb an average r^2^ level of 0.28 ± 0.18 was observed, and at this distance, 35% of the comparisons between SNPs pairs showed a r^2^ ≥ 0.3 (Supplementary Table S1). On the other hand, the BON breed showed an average r^2^ level of 0.26 ± 0.17, and ca. 34% of the comparisons between SNPs pairs show an r^2^ ≥ 0.3. Finally, at 1 Mb distances the ROMO breed shows LD levels of 0.23, while the BON breed shows consistently lower LD levels, with an average r^2^ level of 0.19 at a distance of 1 Mb between pairs of SNPs (Table S1).

## Discussion

On average the MAF for both breeds was 0.27 ± 0.14, with the highest SNPs proportion (23% for BON and 24% for ROMO) showing high MAF values (> 0.4) (Figure 1). These results are consistent with the values reported for other taurine breeds (McKay *et al.*, 2007; Matukumalli *et al.*, 2009; Pérez O’Brien *et al.*, 2014a). However, these were higher compared to average MAF values found in indicine breeds (*Bos indicus*), i.e. between 0.19 and 0.20 (Silva *et al.*, 2010; Espigolan *et al.*, 2013; Pérez O’Brien *et al.*, 2014a). Usually indicine breeds have an opposite trend in MAF levels compared to taurine cattle (*Bos taurus*), with a higher proportion of low allele frequencies (< 0.2) (Gibbs *et al.*, 2009; Matukumalli *et al.*, 2009; Villa-Angulo *et al.*, 2009; Pérez O’Brien *et al.*, 2014b). This has been associated with a greater genetic diversity in indicine populations obtained from sequencing data (Gibbs *et al.*, 2009; Murray *et al.*, 2010). Likewise, this could also be attributed to the fact that the results obtained with the BeadChip BovineSNP50K_v2 used *Bos taurus* breed sequence data, which is genetically distinct from the *Bos indicus* breeds. Hence, the ascertainment bias leads to a higher proportion of low-MAF SNPs in *Bos indicus* breeds. According to Khatkar *et al.* (2008), the MAF threshold directly affects the LD distribution and extent within the population, since there is a significant association between high levels of LD and a higher proportion of SNPs with high MAF values, especially at short distances (Uimari *et al.*, 2005; Sargolzaei *et al.*, 2008; Pérez O’Brien *et al.*, 2014a).

The LD decay analysis of up to 200 kb using 1 kb windows is shown in Figure 3a,b for both breeds evaluated. A rather erratic behavior in the LD decay was observed in the first 20 kb, probably due to a low number of comparisons established between SNPs pairs with distances less than or equal to 20 kb (Table S1). These results suggest that using the set of SNPs contained in the BovineSNP50K_v2 chip, there is no consistency in the LD levels that can be expected for genomic distances of less than 20 kb. These results are however, consistent with those reported by Pérez O’Brien *et al.* (2014a), on a LD analysis conducted in different taurine and indicine breeds. Moreover, in that same study they found that in order to evaluate the LD on a short distance, it is more efficient to use the high-density BovineHD BeadChip® (Illumina Inc., 2010) that reaches an average distance between SNPs of ~ 5 kb compared to the BovineSNP50K_v2 that reaches an average distance between SNPs of ~ 49 kb (Matukumalli *et al.*, 2009).

Authors such as Kruglyak (1999), Meuwissen *et al.* (2001) and Ardlie *et al.* (2002) reported as useful levels of LD r^2^ values larger than 0.3, and this was considered as the minimum required level to develop reliable association studies and to obtain accurate genomic predictions. In this study the ROMO breed presented an average value of r^2^ = 0.3 up to a distance of 100 kb, while the BON breed showed a faster decay, reaching the same value up to a distance of 70 kb. These results differ from the average LD levels reported in other taurine breeds by Pérez O’Brien *et al.* (2014a), which showed that taurine breeds such as Angus, Holstein, Brown Swiss and Fleckvieh reach an average of r^2^=0.3 up to a distance of 40 to 50 kb, while indicine breeds as Gyr and Nelore show a faster LD breakdown, reaching the same r^2^ value up to a distance of about 20 kb. Similar results were found in other taurine dairy and beef breeds that reached similar r^2^ values for distances less than or equal to 30 kb (Bolormaa *et al.*, 2011; Larmer *et al.*, 2014). However, in Holstein cattle in North America (Canada and the United States) Sargolzaei *et al.* (2008) found optimal LD values (r^2^ ≥ 0.3) at distances close to 100 kb, which is similar to our results. On the other hand, at distances of 1 Mb the ROMO breed showed LD levels of 0.23, while the BON breed showed an average r^2^ levels of 0.19 (Table S1), and these values differ from those reported for other cattle breeds that generally show r^2^ levels below 0.1 at distances of 1 Mb (Gautier *et al.*, 2007; McKay *et al.*, 2007; de Roos *et al.*, 2008; Villa-Angulo *et al.*, 2009; Qanbari *et al.*, 2010; Lu *et al.*, 2012; Pérez O’Brien *et al.*, 2014a).

The highest LD level found in the Creole breeds assessed was due to the fact that the individuals evaluated in this study belong to a conservation core population of Creole cattle breeds in Colombia (Vásquez *et al.*, 2007), which are related to a small effective population size. However, LD studies in livestock populations have shown that the LD is much more extensive in these populations than in other species (e.g. humans), and this is mainly due to a smaller effective population size, and in some circumstances, due to stronger selection pressure that has recently been carried out in livestock populations (McRae *et al.*, 2002; McKay *et al.*, 2007; Khatkar *et al.*, 2008). Yet, an exception to this effect/condition in livestock animals is found in rabbits, where the LD declines sharply at short distances due to their rich genetic variability and large effective population size (Carneiro *et al.*, 2014).

Since the average distance between markers that has been reported for the 50 K chip is 49.4 kb (Illumina Inc. 2016), the LD levels found in this study suggest that using the BovineSNP50K_v2 chip as genotyping platform, it is possible to have an adequate coverage throughout the breeds’ genome. In this way, the effect of most QTL related with productive traits would be captured, and an adequate accurate in genomic analysis (GWAS, GS) would be ensured (Kizilkaya *et al.*, 2010). This premise has also been supported in other studies developed in dairy taurine populations, which have shown that the density increase using the 50 K chip compared to using the HD chip has resulted in only increasing slightly the accuracy of genomic predictions (Erbe *et al.*, 2012; Su *et al.*, 2012; VanRaden *et al.*, 2013). Thus, according to Pérez O’Brien *et al.* (2014a) this impact ought to be higher for indicine populations with lower LD levels in shorter distances.

The highest LD levels observed in BON and ROMO breeds compared to reports for similar LD distances (kb) in other cattle breeds, can be related to one or more of the following factors: a higher ancestral relatedness (Purfield *et al.*, 2012), a historically smaller effective population size (founder effect) (Villa-Angulo *et al.*, 2009), or a recent population reduction due to a bottleneck event and genetic drift (Reich *et al.*, 2001; Purfield *et al.*, 2012), which probably occurred in these Creole breeds. In fact, during the last decades these have registered a strong population reduction due to the import of selected and other exotic breeds adapted to tropical conditions (indicine breeds), which has resulted in the widespread displacement of the local Creole cattle. The increase of LD levels can also be a result of the selection pressure in search of productive traits (Thévenon *et al.*, 2007). However, the selection pressure on Creole cattle populations has been minimal (Martínez *et al.*, 2011).

Various authors have proven that effective population sizes have an inverse relationship with LD levels, so usually racial groups with large effective population sizes have lower LD values (Sölkner *et al.*, 1998; Khatkar *et al.*, 2008; Pausch *et al.*, 2013). This condition has been evidenced in indicine breeds which are characterized by having a large effective population size, with a faster LD decay, reaching an average r^2^ value of ≥ 0.3 for a distance of up to 20 kb. On the contrary, taurine breeds have smaller effective population sizes with optimal LD values (r^2^ ≥ 0.3) at distances between 40 to 50 kb (Pérez O’Brien *et al.*, 2014a). Moreover, Ferencakovic *et al.* (2013) found that in populations with higher inbreeding levels as dairy breeds, the LD is stronger. The results of our study indicate that BON and ROMO Creole cattle show high LD values due to a lower effective population size, but also possibly associated with a decrease of the genetic variability within these populations.

In GWAS the proximity and the existence of LD among the markers used will increase the probability of finding QTLs and chromosomal regions of smaller physical size that are less affected by crossing over (Pérez O’Brien *et al.*, 2014a). Many QTLs have segregated exclusively in one population meanwhile others can be shared across different breeds. However, when considering the validity of the results among breeds it is important to remember the differences in LD that exists among different breeds, even in cases where there are similar levels of LD with the possibility to find different LD phases in each breed (Bolormaa *et al.*, 2013). Previous studies have found that the LD phase to be preserved among cattle breeds at only small distances (less than 10 kb) (de Roos *et al.*, 2008) might differ completely between cattle subspecies (Bolormaa *et al.*, 2013). This implies that the association of QTLs and genetic markers through different breeds is only likely to remain when the marker is very close to the QTL, and only for races of the same subspecies (Pérez O’Brien *et al.*, 2014a). The Colombian cattle breeds share a similar origin and have close genetic distances among them (Martínez *et al.*, 2011). Moreover, according with the results from the present study, these breeds have similar LD levels, and therefore, it could be expected that these breeds also share common SNP’s and QTLs of economic importance.

## Conclusion

In BON and ROMO Creole breeds a high proportion of SNPs (45%) with high values of MAF (≥ 0.3) were found, and this may be related with high LD levels found in these populations. These values were superior to those reported in other taurine breeds and reached optimal LD levels (r^2^ ≥ 0.3) in a distance of up to 70 kb for the BON breed, and 100 kb for the ROMO breed, which is possibly associated with several issues: their status as a closed population, having a reduced effective population size, and being subject to a limited occurrence of recent genetic introgression events. The LD levels and allele frequencies distribution found in this study suggests that using the BovineSNP50K_v2 chip as a genotyping platform can result in an adequate coverage throughout the genome for this type of Creole cattle. This in turn may allow capturing the effect of most of the QTLs associated with productive traits and in this way an adequate prediction capacity in the genomic analysis (GWAS, GS) can be ensured.

## Supplementary material

The following online material is available for this article:

Table S1 - Linkage disequilibrium (r^2^) between pairs (N) of SNPs across all autosomes.
