# Supplementary material for: Linkage disequilibrium levels and allele frequency distribution in Blanco Orejinegro and Romosinuano Creole cattle using medium density SNP chip data
Source: Genet Mol Biol. 2018 Apr-Jun;41(2):426–33. doi: 10.1590/1678-4685-GMB-2016-0310 (PMC6082240; doi:10.1590/1678-4685-GMB-2016-0310)
Supplement: Supplementary file 1 [file 1415-4757-GMB-41-02-426-s001.pdf]

Supplementary Material to "Linkage disequilibrium levels and allele frequency distribution in Blanco Orejinegro and Romosinuano Creole cattle using medium density SNP chip data"

Table S1 - Linkage disequilibrium (r^2) between pairs (N) of SNPs separated by different distances across all autosomes.

Distance between SNP (kb)	N		Mean r^2 ± SD		Median		% $r^2 \geq 0.3$[1]	
	ROMO	BON	ROMO	BON	ROMO	BON	ROMO	BON
0 a 10	157	147	0.45±0.32	0.50±0.33	0.29	0.30	48	50
10 a 19	352	368	0.39±0.26	0.43±0.28	0.27	0.32	45	54
20 a 29	4818	4534	0.40±0.27	0.41±0.28	0.28	0.30	47	50
30 a 39	3706	3503	0.37±0.25	0.38±0.26	0.27	0.27	44	46
40 a 49	3106	2930	0.35±0.24	0.35±0.24	0.26	0.26	44	44
50 a 59	3149	2871	0.34±0.24	0.33±0.24	0.25	0.25	40	41
60 a 69	3024	2832	0.32±0.22	0.32±0.22	0.24	0.24	39	40
70 a 79	2932	2694	0.32±0.22	0.30±0.21	0.24	0.23	39	37
80 a 89	2941	2564	0.31±0.21	0.30±0.20	0.23	0.23	37	36
90 a 100	2785	2467	0.29±0.20	0.29±0.20	0.23	0.22	34	34
100 a 200	20829	13429	0.28±0.18	0.26±0.17	0.23	0.22	35	34
200 a 300	18978	11421	0.25±0.15	0.22±0.14	0.22	0.20	31	26
300 a 400	18082	10402	0.25±0.15	0.21±0.12	0.21	0.19	29	22
400 a 500	17448	9677	0.24±0.14	0.20±0.11	0.21	0.19	27	20
500 a 600	16870	9452	0.24±0.14	0.20±0.11	0.20	0.18	27	19
600 a 700	16595	9168	0.23±0.13	0.20±0.10	0.20	0.18	26	18
700 a 800	16364	8872	0.23±0.13	0.19±0.10	0.20	0.18	26	17
800 a 900	16038	8694	0.23±0.13	0.19±0.10	0.20	0.18	25	16
900 a 1000	15892	8530	0.23±0.13	0.19±0.09	0.20	0.18	24	16

SNP: single-nucleotide polymorphism; SD: Standard deviation. [1]Percentage of SNP pairs with $r^2 \geq 0.3$.
